# Oromandibular-limb Hypogenesis Syndrome Type II C: A Rare Case

**DOI:** 10.5681/joddd.2010.033

**Published:** 2010-12-21

**Authors:** Renita Lorina Castelino, Shishir Ram Shetty, Subhas Babu G, Kumuda Arvind Rao H T

**Affiliations:** ^1^ Post-graduate Student, Department of Oral Medicine and Radiology, A.B Shetty Memorial Institute of Dental Sciences, Mangalore, India; ^2^ Assistant Professor, Department of Oral Medicine and Radiology, A.B Shetty, Memorial Institute of Dental Sciences, Mangalore, India; ^3^ Professor, Department of Oral Medicine and Radiology, A.B Shetty Memorial Institute of Dental Sciences, Mangalore, India; ^4^ Post-graduate Student, Department of Oral Medicine and Radiology, A.B Shetty Memorial Institute of Dental Sciences, Mangalore, India

**Keywords:** Hypoglossia, peromelia, teratogenic

## Abstract

The oromandibular-limb hypogenesis syndrome comprises a group of anomalies which simultaneously affect the mandible, tongue, and maxilla with or without reductive limb anomalies. It is characterized by failure of development of the intraoral region and distal extremities. Multiple and variable deformities of the mandible, maxilla and tongue may occur in combination with a variety of limb defects. The wide range of presentation and combination of anomalies make classification difficult. They usually feature primarily in sporadic case reports because of their low incidence. The genetic origin of this syndrome is uncertain. It is congenital and there seems to be no sex predilection. The key radiographic features are retruded mandible, impacted teeth and malformed phalanges. When compared to available literature, frequently reported features like hypodontia, hypoglossia, microstomia, protruded maxilla and limb anomalies were present in our case. The case presented here is one of the rarest subtypes of this rare syndrome.

## Introduction

Oromandibular-limb hypogenesis syndromes (OLHS) represent a group of rare conditions characterized by congenital malformations involving multiple sites such as the tongue, mandible, and limbs.^1^ In 1971, Hall classified OLHS into 5 major types and according to this, the case report presented here falls under type II C, which is hypoglossia-hypodactylomelia syndrome.^2,3^ The hypoglossia-hypodactyly syndrome, the Moebius syndrome, the Hanhart syndrome, the Charlie M syndrome and OMLH are possibly variants of the same condition, and it is often difficult to define the phenotypic boundaries between them.^4,5^ There is considerable overlap between these syndromes gathered under the term OLHS, with a marked variability of face and limb anomalies as well as other additional malformations.^1^ Limb deficiencies are major congenital malformations and can result from a number of etiological factors.^6^ Heat-induced vascular disruption has been considered as one of the etiological factors for these syndromes.^7,8^ Apart from this, teratogenic aetiology has also been implicated.^4,9^ The genetic origin of these syndromes is uncertain.^10^ However, most of the cases are sporadic.^6^ Forty-seven cases of hypoglossia-hypodactylia (Type I A) syndrome have been reported before 1990.^11^ The exact incidence of Type II C of OLHS could not be traced from the available literature. This case is reported to highlight the clinical and radiological presentation of this rare syndrome. The radiographic features consisted of retruded mandible in the lateral cephalograms, impacted teeth in orthopantomogram and malformed phalanges in wrist radiographs.^12^

The most accepted classification was proposed by Hall in 1971.^4^

## Case Report

A 23-year-old male patient reported to our institute with a chief complaint of speech difficulties since birth. The patient also complained of microstomia, malaligned teeth and difficulty in walking. The patient had no difficulty in breathing and swallowing. The patient was the second child out of two children. There was no history of con-sanguineous marriage between the parents. There was no history of similar findings in the family. The patient reported a history of drug intake by his mother during the third month of her pregnancy. On general examination, the patient had an unusual gait due to peromelia of lower limbs. The patient was well oriented to time and place.

On extraoral examination, the patient exhibited peromelia of upper and lower limbs (Figure 1a,b), microstomia, protruded upper anterior teeth, retruded chin, incompetent lips and fusion of the lower alveolar mucosa with the cutaneous part of the lower lip. Intraoral examination revealed constricted maxillary and mandibular arches, absence of lower labial sulcus due to the fusion of lower lip to the alveolar mucosa (Figure 1c), hypoglossia (Figure 1d), hypodontia and root stumps in relation to the teeth #36, #43 and #46. The orthopantomograph revealed thinning of both the condylar heads, asymmetry between the right and left mandibular bodies and increased gonial angle (Figure 2a). Lateral cephalogram revealed retruded mandible and protruded upper maxillary teeth (Figure 2b). The radiograph of the hand showed absence of carpal bones (Figure 2c). The radiograph of the leg showed hypoplastic tarsal bones skeletal pattern (Figure 2d). After a thorough evaluation by a team of medical specialists, we arrived at a final diagnosis of oromandibular-limb hypogenesis syndrome Type II C.

Figure 1. 
Clinical photograpgh of the patient showing peromelia of upper limbs (a); hypodactylia of lower limbs (b); constricted maxillary arch and fusion of the cutaneous part of the lower lip to the alveolar mucosa (c); clinical photograph showing hypoplastic tongue (d).

**a:**
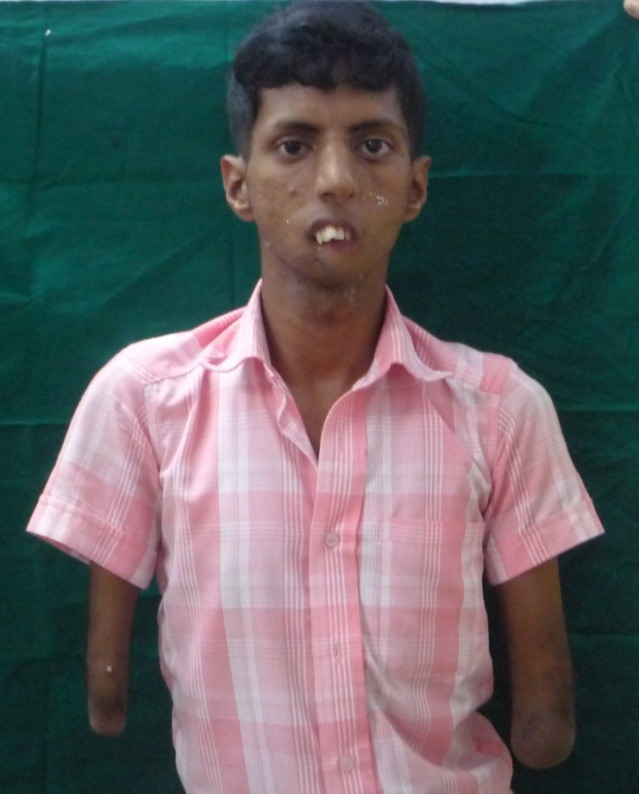

**b:**
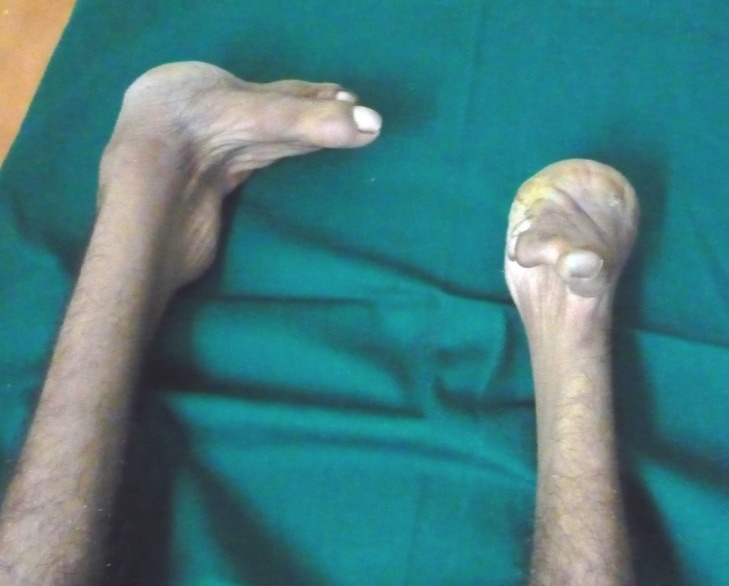

**c:**
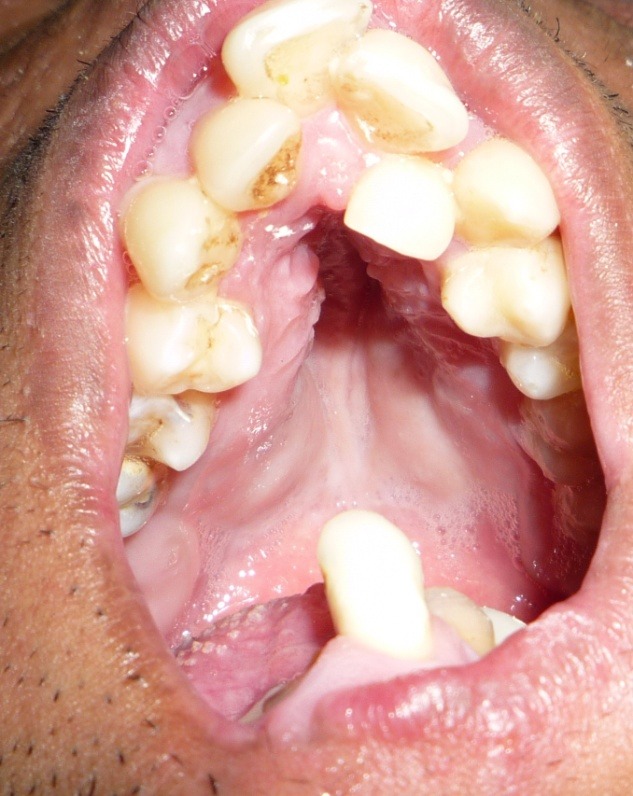

**d:**
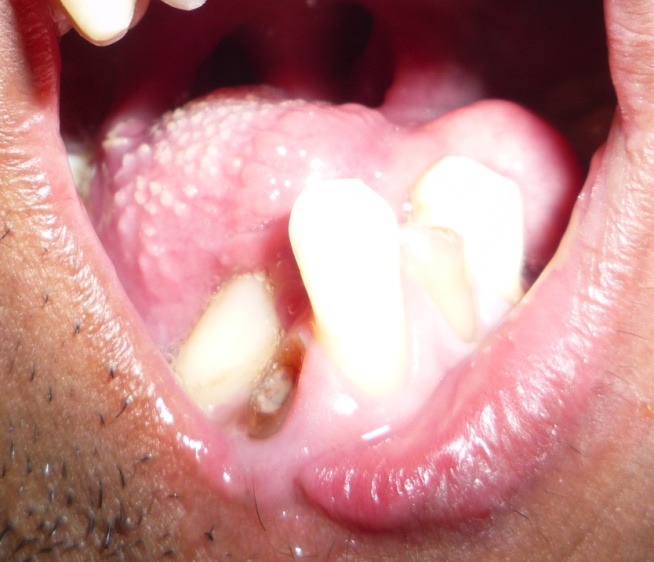

Figure 2. 
Orthopantomograpgh showing thinning of both the condyles (a); lateral cephalogram showing retruded chin (b); radiograph of the hand showing absence of phalanges (c); radiograph of the leg showing abnormal skeleton pattern (d).

**a:**
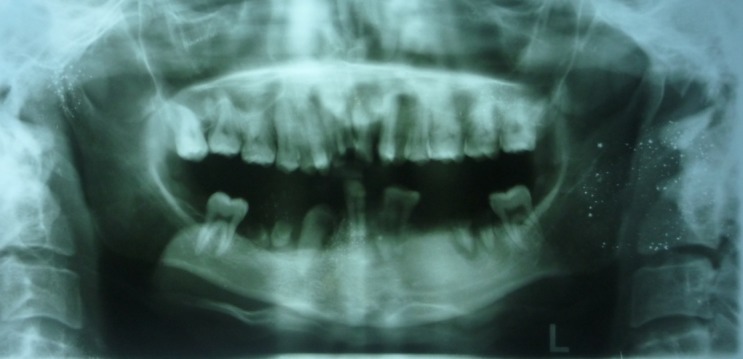

**b:**
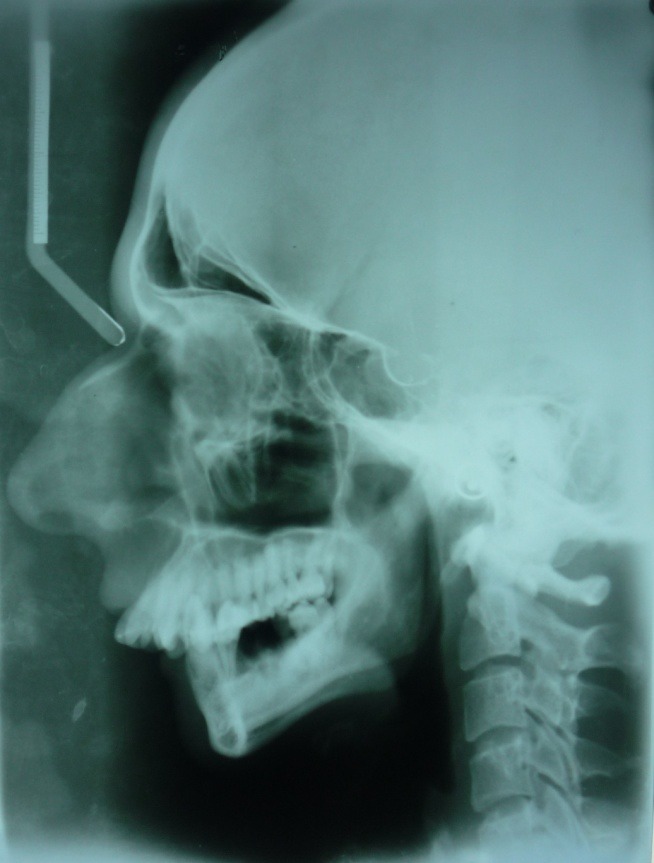

**c:**
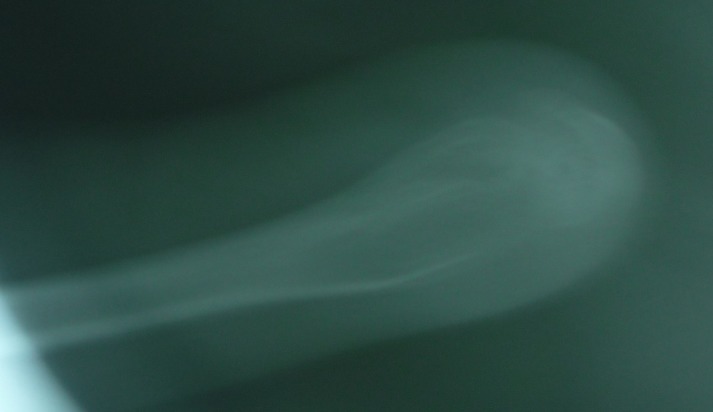

**d:**
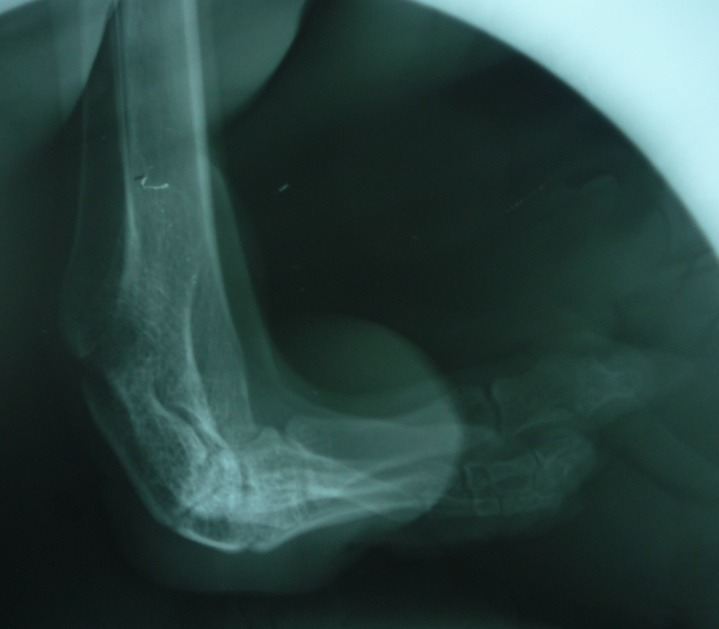

**Table 1:** Hall’s classification of oromandibular-limb hypogenesis syndrome

Type I	A: hypoglossia B: aglossia
Type II	A: hypoglossia-hypodactylia B: hypoglossia-hypomelia C: hypoglossia-hypodactylomelia
Type III	A: glossopalatine ankylosis (Ankylossum Superius syndrome) B: with hypoglossia C: with hypoglossia-hypodactylia D: with hypoglossia-hypomelia E: with hypoglosia-hypodactylomelia
Type IV	A: intraoral bands and fusion B: with hypoglossia C: with hypoglossia-hypodactylia D: with hypoglossia-hypomelia E: with hypoglossia-hypodactylomelia
Type V	A: Hanhart syndrome B: Charlie M syndrome C: Pierre-Robin syndrome D: Mobius syndrome E: amniotic band syndrome

## Discussion

Our patient presented with extraoral features, including convex profile, micrognathic mandible with relative maxillary prognathism and intraoral features, including microstomia, hypodontia, hypoglossia, and constricted maxillary and mandibular arches. Our patient also presented with upper and lower limb anomalies. A brief comparison of available literature with our case findings is discussed below.

Our patient presented with features like convex profile, retruded mandible and proclined maxillary anterior teeth, consistent with features reported by Wadhwani et al^12^ in a case report; however, they reported about bilaterally impacted teeth which were not observed in our case. However, in rare instances protruded lower jaw has also been reported.^13^

The case presented here also showed upper and lower limb anomalies similar to the findings reported by Wadhwani et al and Figuero et al.^12, 13^

It was first reported by Rosenthal^14^ in 1932 as aglossia congenita. The OLHS is a rare complex of jaw and limb defects with unclear aetiology.^12^There seems to be no sex predilection.^14^ However, the proposed aetiology is heredity, maternal hyperthermia and positive drug history during pregnancy. This syndrome sometimes presents with cranial nerve palsies (sixth and seventh).^6^ Hermann et al^15^ analysed OLHS cases and found that there was severity of upper limb involvement, especially malformation of the feet, but they did not find cranial nerve palsies and this was significant in differentiating the cases. The group of patients with cranial nerve palsies included some with limb defects similar to those observed in Hanhart syndrome and others with Poland anomaly; finally, cases with cranial nerve palsies without limb involvement were documented.^15^ No evidence of cranial nerve palsy was observed in the case presented here. Past medical history for exposure of the mother to drugs during pregnancy was positive in a case report.^14^ A similar history was reported in our case. Multiple site involvement and the wide range and combination of anomalies make classification difficult.^16^ There is overlap and similarity between different syndromic entities among similarities with OLHS, including a long list of syndromes like Moebius syndrome, hypoglossia hypodactylia syndrome, Hanhart syndrome, glossopalatine ankylosis syndrome, limb deficiency, splenogonadal fusion syndrome, and Charlie M syndrome. All are very uncommon except for Moebius syndrome.^16^ These groups of syndromes require a long-term and multidisciplinary approach.^17^The case reported here is a rare syndrome with multiple site involvement. The treatment includes replacement of upper and lower limbs with prostheses, correction of malocclusion and speech therapy. Similar treatment modalities were suggested to the patient but the patient could not afford them.

## Conclusion

The case presented here is a rare subtype of oromandibular-limb hypogenesis syndrome with oral manifestations, including hypodontia, hypoglossia, retruded chin and malaligned teeth along with limb anomalies. Almost all cases reported to date are seemingly sporadic.

## References

[1] Brockmann K, Backes H, Auber B, Kriebel T, Stellmer F, Zoll B (2009). Overlap of Moebius and oromandibular limb hypogenesis syndrome with gastroschisis and pulmonary hypoplasia. Am J Med Genet A.

[2] Rasool A, Zaroo M I, Wani A H, Darzi M A, Bashir S A, Bijli A H and Rashid S. Isolated aglossia in a six-year-old child presenting with impaired speech: a case report. Cases Journal 2009, 2:7926 10.4076/1757-1626-2-7926 PMC2769388 19918438

[3] Jang GY, Lee KC, Choung JT, Son CS, Tockgo YC (1997). Congenital aglossia with situs inversus totalis. JKMS.

[4] Hall BD: Aglossia-adactylia. Birth Defects Orig Artic Ser 1971, 7:233-6. 5173209

[5] Hanhart E: Ueber die Kombination von Peromelie mit Mikrognathie, ein neues Syndrom beim Menschen, entsprechend der Akroteriasis congenita von Wriedt und Mohr beim Rind. Arch Klaus-Stift Ver 1950, 25:531-43.

[6] Kaissi A A, Safi H, Ghachem M B , Hendaoui L Chehida F B. Aglossia-adactylia sequence and Moebius syndrome involvement. African Journal of oral Health 2005,2: 37-41

[7] Superneau DW, Wertelecki W (1985). Brief clinical report: Similarity of effects - Experimental hyperthermia as a teratogen and maternal febrile illness associated with oromandibular and limb defects. Am. J. Med. Genet.

[8] Robinow M (1978). Discordance in monozygotic twins for aglossia adactylia, and possible clues to the pathogenesis of the syndrome. BDOAS.

[9] Bokesoy I, Aksuyek C, Deniz E (1983). Oromandibular limb hypogenesis/Hanhart's syndrome: possible drug influence on the malformation. Clin Genet.

[10] Chicarilli ZN, Polayes IM (1985). Oromandibular limb hypogenesis syndromes. Plast Reconstr Surg.

[11] Bagnulo MA, Ferreira SL, Sanchez Z, Cangialosi TJ (1999). Hypoglossia- Hypodactylia Type IA: A Case Report. Columbia Dental Review.

[12] Wadhwani P, Mohammad S, Sahu R (2007). Oromandibular limb hypogenesis syndrome, type IIA, hypoglossia-hypodactylia: a case report. J Oral Pathol Med.

[13] Figueroa A, Pruzansky S (1982). Terminal transverse defects with orofacial malformations (TTV-OFM) : Case Report with Mandibular Prognathism and Submucous Cleft Palate. Cleft palate journal.

[14] Perks T J, Van Der Walt, Levin AI, Graewe FR (1998). An unusual case of oromandibular-limb hypogenesis syndrome. Eur J Plast Surg.

[15] Herrmann J, Pallister PD, Gilbert EF (1976). Studies of malformation syndromes of man XXXXIB. Nosologic studies in the Hanhart and the Moebius syndrome. Eur J Pediatr.

[16] Preis S, Majewski F, Hantschmann R, Lenard HG, Schumacher H (1996). Goldenhar, Moebius and hypoglossia-hypodactyly anomalies in a patient: syndrome or association?. Eur J Pediatr.

[17] Alexander R, Freidman JS, Eichen MM (1992). Oromandibular limb hypogenesis syndrome; type II A, hypoglossia-hypodactylia - report of a case. Br J Oral Maxillofac Surg.

